# Revealing the mechanisms of RAC3 in tumor aggressiveness, the immunotherapy response, and drug resistance in bladder cancer

**DOI:** 10.3389/fonc.2024.1466319

**Published:** 2024-09-16

**Authors:** Hanyuan Gao, Yanru Qiu, Xueqin Zheng, Tianwen Xu, Guangjian Liu

**Affiliations:** ^1^ Department of Nephrology, The Second Affiliated Hospital of Fujian Medical University, Quanzhou, China; ^2^ Department of Oncology, The Second Affiliated Hospital of Fujian Medical University, Quanzhou, Fujian, China

**Keywords:** bladder cancer, Rac3, tumor aggressiveness, immunotherapy response, drug resistance

## Abstract

**Background:**

Bladder cancer (BLCA) is a prevalent urinary tract malignancy with a high propensity for recurrence and chemoresistance. The molecular mechanisms underlying its progression and response to therapy have not been fully elucidated.

**Methods:**

We conducted a multifaceted analysis, integrating immunohistochemical (IHC) staining, bioinformatics evaluation using TCGA and CCLE databases, and *in vitro* assays using the BLCA cell lines 5637 and T24. RAC3 expression was assessed relative to clinical and pathological features. Functional enrichment analyses and gene set enrichment analysis (GSEA) were performed to identify associated biological processes and pathways. The impacts of RAC3 on cell proliferation, migration, invasion, and the immune microenvironment were evaluated using siRNA knockdown, CCK-8, Transwell, wound healing and colony formation assays.

**Results:**

Elevated RAC3 expression was significantly correlated with an advanced tumor stage, lymph node metastasis, and poor prognosis for BLCA patients. The functional enrichment analysis implicated RAC3 in immune cell infiltration and immune checkpoint mechanisms. Notably, RAC3 knockdown significantly reduced the proliferative, migratory, and invasive capabilities of BLCA cells. These effects were reversed by the overexpression of RAC3. Additionally, RAC3 expression was linked to chemoresistance, with high RAC3 expression predicting resistance to certain therapeutic agents. The TIDE algorithm indicated that RAC3 expression could be a predictive biomarker for the immunotherapy response.

**Conclusion:**

RAC3 was identified as a potential therapeutic target and biomarker of BLCA, as its expression significantly influenced tumor progression, the immune response, and chemosensitivity. Targeting RAC3 may provide a novel strategy for the management of BLCA, particularly for patients resistant to conventional therapies. Further research is essential to elucidate the detailed mechanisms of RAC3 in BLCA and explore its clinical application in precision medicine.

## Introduction

Bladder cancer, a prevalent urinary tract malignancy, is almost exclusively linked to the urothelium, with 90% of cases stemming from this tissue (1). The prognosis for bladder cancer patients with lung or bone metastases is sobering, typically with a median survival period of approximately 15 months. The 5-year survival rate for such patients is generally between 5% and 10% (2, 3). These findings suggest that the survival prospects for patients with advanced bladder cancer are quite bleak. Identifying more efficacious treatments is of paramount importance to increase the long-term survival of these patients.

Recent research has shown that targeted therapies and immunotherapies might provide benefits to certain patients (4–8). Given its immunogenic nature, bladder cancer may be more likely to provoke a pronounced immune response, offering potential therapeutic advantages (9). However, similar to other types of cancer, drug resistance remains the greatest challenge for these therapeutic approaches (10). Moreover, patients with low PD-L1 expression often find single-agent immunotherapy to be less effective, while combination chemotherapy is more likely to provide clinical advantages. Platinum-based chemotherapy remains the primary treatment option for patients with recurrent and advanced stages of bladder cancer, but its limited effectiveness and the development of drug resistance are significant contributors to disease progression (11, 12). The mechanisms underlying drug resistance are not yet fully understood, and improving treatment outcomes while reducing the incidence of resistance is of critical clinical importance. Research indicates that alterations in the immune microenvironment are linked to the emergence of cisplatin resistance (13, 14). In addition, immunotherapy has the potential to reshape the tumor immune microenvironment. Consequently, the combination of immunotherapy with chemotherapy may synergistically enhance their therapeutic effects and even potentially counteract cisplatin resistance.

The Rho family of guanosine triphosphatases (GTPases) act as indispensable regulators within the intricate network of cell signaling pathways and cytoskeletal dynamics (15, 16). Their influence extends to pivotal cellular processes such as adhesion, morphology, migration, and the cell cycle, underscoring their importance in a diverse array of cell types. These enzymes have the remarkable ability to promote tumor progression (17). RAC3, a Rho subfamily Ras protein, is a gene that encodes a small GTPase with a crucial role in orchestrating the actin cytoskeleton and the complex cascade of intracellular signal transduction (18, 19). The discovery that RAC3 may function as an oncogene highlights its influence on cancer development, suggesting that it could be a key player in the intricate dance of oncogenesis. According to recent findings, a pronounced association between RAC3 expression and immune cell infiltration exists in the tumor microenvironment, suggesting that RAC3 might function as an indicator of the immune status and is capable of influencing the immune microenvironment and tumor immune response (20). Consequently, RAC3 may be a novel target for immunotherapeutic interventions. Additionally, RAC3 contributes to the development of chemoresistance in tumors. Wang et al. (21) revealed that the genetic silencing of RAC3 in bladder cancer cells increased their susceptibility to gemcitabine, suggesting that RAC3 could play a role in the progression of chemotherapy resistance in bladder cancer by modulating the immune microenvironment. The development of an optimal molecular signature, distinguished by its chemical responsiveness and immune attributes, is crucial for an accurate risk analysis and the personalized determination of treatment strategies, potentially leading to improved outcomes and clinical results for high-risk patients with bladder cancer.

Our study demonstrates that RAC3 is highly expressed in bladder cancer and is correlated with the clinical and pathological features of patients, suggesting that RAC3 may facilitate the progression of bladder cancer. Building on these findings, we validated the regulatory role of RAC3 in the clinical and pathological characteristics, immune microenvironment, and chemoresistance of bladder cancer at the bioinformatics level. Furthermore, at the cellular level, we confirmed the impacts of RAC3 on the proliferation, migration, invasion, and chemokine expression of bladder cancer cells. Our research indicates that RAC3 potentially modulates the expression of chemokines, reshaping the immune microenvironment and mediating the development of chemotherapy resistance in bladder cancer.

## Materials and methods

### Immunohistochemical staining

Sixty sets of bladder urothelial carcinoma (BLCA) tissues and their adjacent normal tissues were collected from patients who underwent surgical resection at The Second Affiliated Hospital of Fujian Medical University between January 2019 and December 2020. All patients had pathologically confirmed BLCA without any history of other cancers and had not received radiotherapy, chemotherapy, or any other targeted treatments prior to surgery. The study received ethical approval from the hospital’s Ethics Committee (No. 2024285). Immunohistochemical (IHC) staining for RAC3 was conducted using a specific monoclonal antibody (Clone: ab124943, Abcam, USA) according to the manufacturer’s guidelines. Two independent pathologists assessed the presence of RAC3 in tumor cells and calculated the average percentage of positively stained cells across five randomly selected fields. A staining threshold of ≥1% was established to define a positive RAC3 result.

### Bioinformatics analysis

The transcriptome data for our study were obtained from TCGA (22), GTEx (23), and CCLE (24) databases and included samples from both neoplastic and nonneoplastic tissues, as well as various cell lines. We performed a detailed intergroup comparison and survival analysis.

### Tumor immune analyses

TCGA-BLCA dataset was employed for assessing various parameters: microsatellite instability (MSI), tumor mutational burden (TMB), the immune context of tumors, and scores reflecting immune cell infiltration. MSI was gauged with the Microsatellite Analysis for Normal Tumor InStability (MANTIS) tool (25). The computation of the TMB was executed with the ‘maftools’ R package, version 2.2.10 (25). The ESTIMATE algorithm was applied to evaluate the immune microenvironment in relation to RAC3 expression in BLCA (26). The ESTIMATE algorithm was used to investigate the link between RAC3 expression levels and the extent of immune cell infiltration in BLCA, including the immune score, ESTIMATE score, and tumor purity, in BLCA samples. For all analyses involving the calculation of rank correlation coefficients, Spearman’s Rho method was the chosen approach.

### Immunotherapy evaluation

RNA-sequencing expression profiles and corresponding clinical information for bladder cancer patients were downloaded from TCGA dataset (https://portal.gdc.com). The potential ICB response was predicted with the TIDE algorithm (27). The R programming language, augmented with the “limma” and “ggpubr” libraries, was used to conduct comparative immunotherapy analyses between groups exhibiting different clinical and pathological characteristics.

### Functional enrichment analysis

To explore the differential pathway enrichment between groups with high and low RAC3 expression, we utilized the “ggplot2” and “GSVA” R packages for KEGG pathway analysis. The GSVA method, which is unsupervised and nonparametric, offered insights into the variation in gene set enrichment among the samples. Additionally, we conducted Gene Set Enrichment Analysis (GSEA) with the c5.go.v7.5.1 hallmark gene sets (28), applying GSEA software version 4.2.3.

### Cell lines and cell culture

Our investigation utilized the bladder cancer cell lines 5637, T24 and the SV-HUC-1 normal uroepithelial cell line, both of which were acquired from Procell Life Science & Technology, Wuhan, China. The cells were cultured in RPMI 1640, McCoy’s 5A or Ham’s F-12K media from Gibco, USA, supplemented with 10% heat-inactivated FBS and antibiotics (100 U/mL penicillin and streptomycin). The culture was maintained in a humidified incubator at 37°C with an atmosphere containing 5% CO2.

### Transfection of small interfering RNAs

RAC3 was targeted by three distinct siRNA constructs, designated siRAC-1, siRAC-2, and siRAC-3, to knockdown its expression, with a nontargeting siRNA serving as the control, all of which were procured from Sangon Biotech in Shanghai, China. The transfection process was facilitated by LipoRNAi reagent, a product from Beyotime, Shanghai, China. Prior to transfection, the 5637 and T24 cells were plated in a 10 cm dish and allowed to grow to 70–80% confluence in complete growth medium. Following transfection, which occurred 18–24 hours after seeding, the cells were cultured in fresh complete medium for 24 additional hours. The experimental group, known as the siRAC group, received siRNAs specific to RAC3, whereas the control group, termed the si-NC group, was transfected with siRNAs targeting a nonspecific sequence. In control group, only the transfection reagent was added without any siRNA. The specific siRNA target sequences are described in detail in **Supplementary Table S1**.

### Establishment of a cotransfection system

We established a cotransfection system in cells by transfecting siRNAs along with an overexpression plasmid (Hanbio, Shanghai, China) to validate the effects of siRNA transfection and perform rescue experiments. The objective was to counteract the effect of siRNA-mediated knockdown on the expression of RAC-3. The specific procedure involved the transfection of the siRNAs with LipoRNAi reagent (Beyotime, Shanghai, China) and the overexpression plasmid with LipoFiter3.0 (Hanbio, Shanghai, China). The 5637 and T24 cells were plated in 10 cm culture dishes and cultured in complete growth medium until they reached 70–80% confluence. Transfection was initiated 24 hours after seeding, and the cells were then further cultured in fresh complete medium for an additional 24 hours. The experimental group, designated the si+ovRAC group, contained a specific overexpression plasmid, the details of which can be found in **Supplementary Figure S1**.

### Quantitative reverse transcription PCR

Total RNA was extracted from 5637 cells and T24 cells using an RNA extraction kit from Beyotime (Shanghai, China) according to the manufacturer’s protocol. The RNA was subsequently reverse transcribed into cDNA using a cDNA synthesis kit from TaKaRa (Tokyo, Japan). For the quantitative gene expression analysis, the cDNA was processed with SYBR Premix Ex Taq reagent from TaKaRa (Tokyo, Japan). The expression of RAC3 was quantified relative to that of the housekeeping gene GAPDH using the 2^-ΔΔCT^ method. The sequences of primers used for gene amplification were provided by Sangon Biotech (Shanghai, China) and are listed in **Supplementary Table S1**.

### Western blotting analysis

The cell lysates were meticulously prepared with lysis buffer (Beyotime Biotechnology, Shanghai, China). Protein concentrations were quantified with a BCA kit, which was also provided by Beyotime. The samples were resolved on SDS−PAGE gels and transferred to a PVDF membrane (Millipore, Bedford, MA, USA). The membrane was blocked with Beyotime’s rapid sealing solution for 30 minutes, followed by an overnight incubation at 4°C with primary antibodies. After three washes with TBST, the membrane was incubated with an HRP-conjugated goat anti-rabbit IgG antibody (Cat No. AS014; ABclonal, Wuhan, China; 1:4000) for 1 hour at room temperature. The immunoreactive bands were visualized using the Clarity Western ECL substrate (Beyotime Biotechnology, Shanghai, China) and captured on autoradiographic film. The antibodies used included those against GAPDH (Cat No. 10494-1-AP; Proteintech, Wuhan, China; dilution, 1:20000) and RAC3 (Cat No. ab124943; Abcam, USA; dilution, 1:5000).

### Cell counting kit-8 assay

The proliferative activity of bladder cancer (BLCA) cells was assessed using the Cell Counting Kit-8 (CCK-8) from Everbright, Inc. (USA). After a 24-hour transfection period, the 5637 and T24 cells were seeded into a 96-well plate at a concentration of 1×10^4 cells/mL. CCK-8 solution was added to the wells, and the plates were incubated at 37°C. At 0, 24, 48, and 72 hours, the optical density (OD) was measured at 450 nm using a spectrophotometer to determine the cell proliferation rate.

### Migration and invasion assays

The migratory and invasive properties of the 5637 and T24 cell lines were evaluated using Transwell assays. At 24 hours post transfection, the BLCA cells were detached with trypsin and suspended at a density of 1×10^5 cells/mL in serum-free RPMI 1640 medium. For the migration assay, 2×10^4 cells in 200 μL of medium were added to the upper chamber of the Transwell system, which faced the lower chamber containing 600 μL of medium supplemented with 10% FBS. For invasion assays, the upper surface of the Transwell membrane was coated with 50 μL of Matrigel from BD Bioscience, USA. Following a 24-hour incubation, the cells were fixed with 4% paraformaldehyde for 30 minutes and stained with crystal violet for 20 minutes. The experimental quantification was based on counting the number of cells in the lower compartment across five random fields to determine the average number of migrated or invaded cells.

### Wound healing assay

The migratory capacity of the cells was assessed through a wound healing assay. Cells at nearly 90% confluence post transfection growing in 6-well plates were scratched with a 200 μL pipette tip. The detachment of nonadherent cells was facilitated by rinsing with PBS. After a subsequent 24-hour incubation in serum-free medium, cells were monitored by capturing images at 24 and 48 hours using a microscope, with attention to consistent fields of view. The migration rate was determined by calculating the wound closure rate with the following formula: wound closure rate = ((initial scratch area at 24 h)-scratch area at 48 h)/initial area at 24 h) * 100%. These assays were repeated a minimum of three times for reliability.

### Colony formation assay

In the experimental setup for the colony formation assay, 1000 cells were plated in each well with 2 mL of RPMI 1640 medium containing 10% FBS and cultured for approximately 7 days at 37°C with 5% CO2 to form colonies. After the incubation period, the cells were washed with PBS, fixed with 4% paraformaldehyde for 15 minutes, and stained with crystal violet for 5 minutes. The colonies were then photographed, and the counts were analyzed statistically to determine the results.

### Statistical analysis

IBM SPSS Statistics version 23.0 software, GraphPad Prism version 8.0 software, and R software were used as statistical tools. The chi-square test was used to analyze categorical data. Clinical patient data are presented as the means ± standard deviations (SDs). Spearman’s correlation coefficients were calculated for the correlation analysis. For the bioinformatics analysis, the Wilcoxon test was used to compare differences between continuous variables. The Kruskal−Wallis test was used to compare differences between multiple groups. For *in vitro* experiments, since a normal distribution was not expected, a nonparametric two-tailed Student’s t test was used to calculate significant results. A p value of <0.05 was considered to indicate statistical significance.

## Results

### RAC3 was highly expressed in BLCA

**Figure 1:**
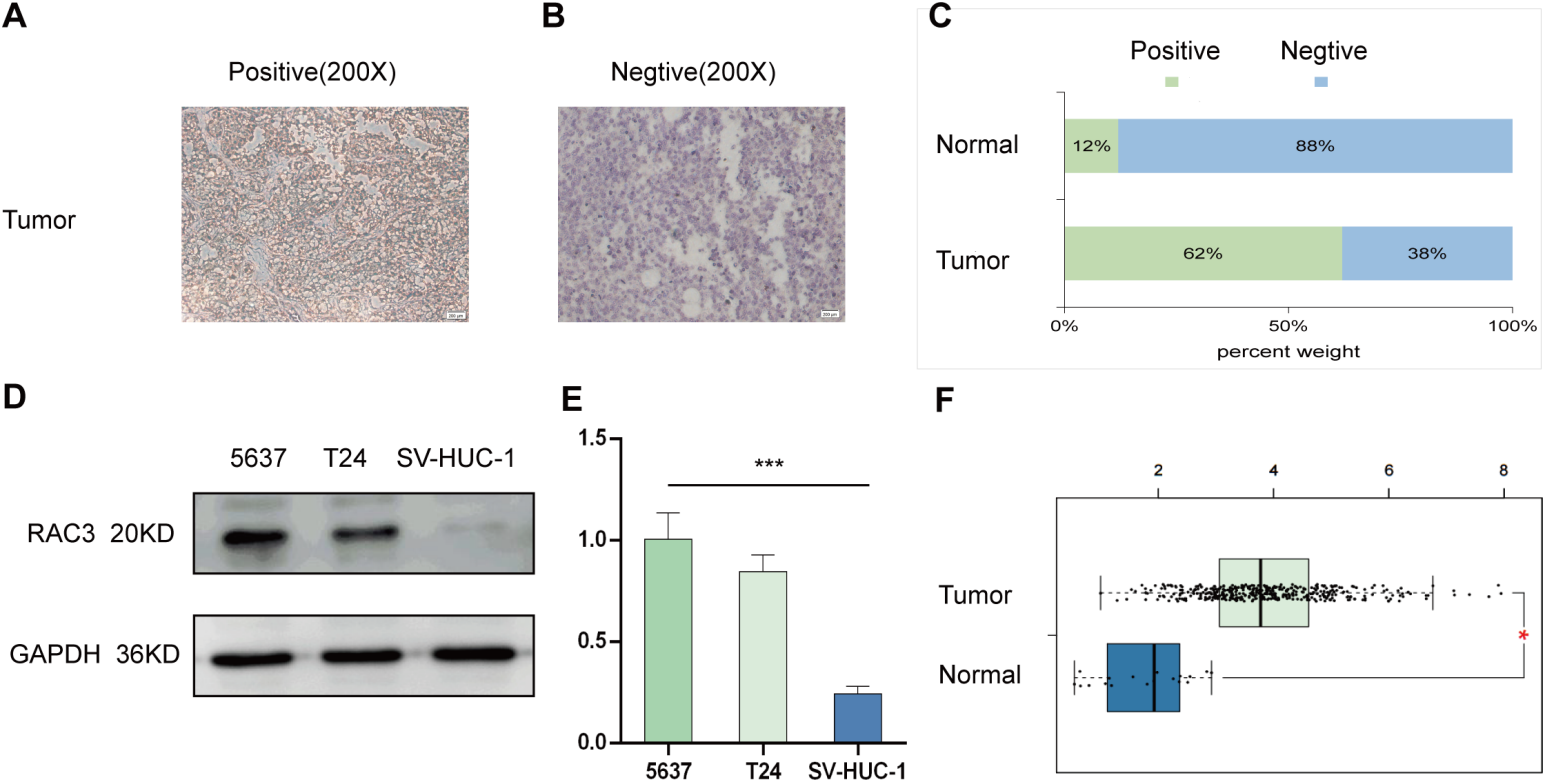
RAC3 expression was elevated in bladder cancer (BLCA). **(A, B)** Immunohistochemistry (IHC) was utilized to examine the presence of RAC3 in positive expression **(A)** and negative expression **(B)**. **(C)** A comparison was made to highlight the variations in RAC3 levels between the cancerous and adjacent normal tissues. **(D)** Western blotting was used to determine RAC3 protein levels in the 5637, T24 and SV-HUC-1 cell lines. **(E)** RAC3 mRNA expression was measured in the 5637, T24 and SV-HUC-1 cell lines using qRT–PCR. **(F)** The expression difference of RAC3 in bladder cancer tumor tissues compared to normal tissues from TCGA database. The experiment was repeated at least 3 times. The symbols * and *** indicate statistical significance at the p<0.05 and p<0.001 levels, respectively.

The role of RAC3 in bladder cancer (BLCA) was explored through a series of analyses focusing on its expression levels in BLCA tissues and cell lines. An immunohistochemical (IHC) study was performed on tissue sections from human BLCA tumors and corresponding normal tissues (**Figures 1A, B**). In a cohort of 60 surgically removed BLCA samples, RAC3 was found to be overexpressed (detectable at >1%) in 62% of the cases, with 37 of 60 samples testing positive, and it was under expressed (detectable at <1%) in 38% of the cases, with 23 of 60 samples testing negative (**Figure 1C**). Our findings indicate a significant increase in RAC3 gene expression within the tumor tissues compared with the surrounding normal tissue samples. We assessed the expression of RAC3 in BLCA cell lines (5637 cells and T24 cells) and a normal uroepithelial cell line (SV-HUC-1 cells) using qRT−PCR and Western blot (**Figures 1D–F**) analyses to further examine this finding. These results were consistent with those of the IHC analysis, which revealed notably lower expression of RAC3 in normal uroepithelial cells (SV-HUC-1 cells) than in BLCA cells (5637 cells and T24 cells). This study indicates that the level of RAC3 expression in bladder cancer tissues is notably elevated compared with that in normal tissues.

### High expression of RAC3 is associated with a poor prognosis and clinicopathological features of BLCA

**Figure 2:**
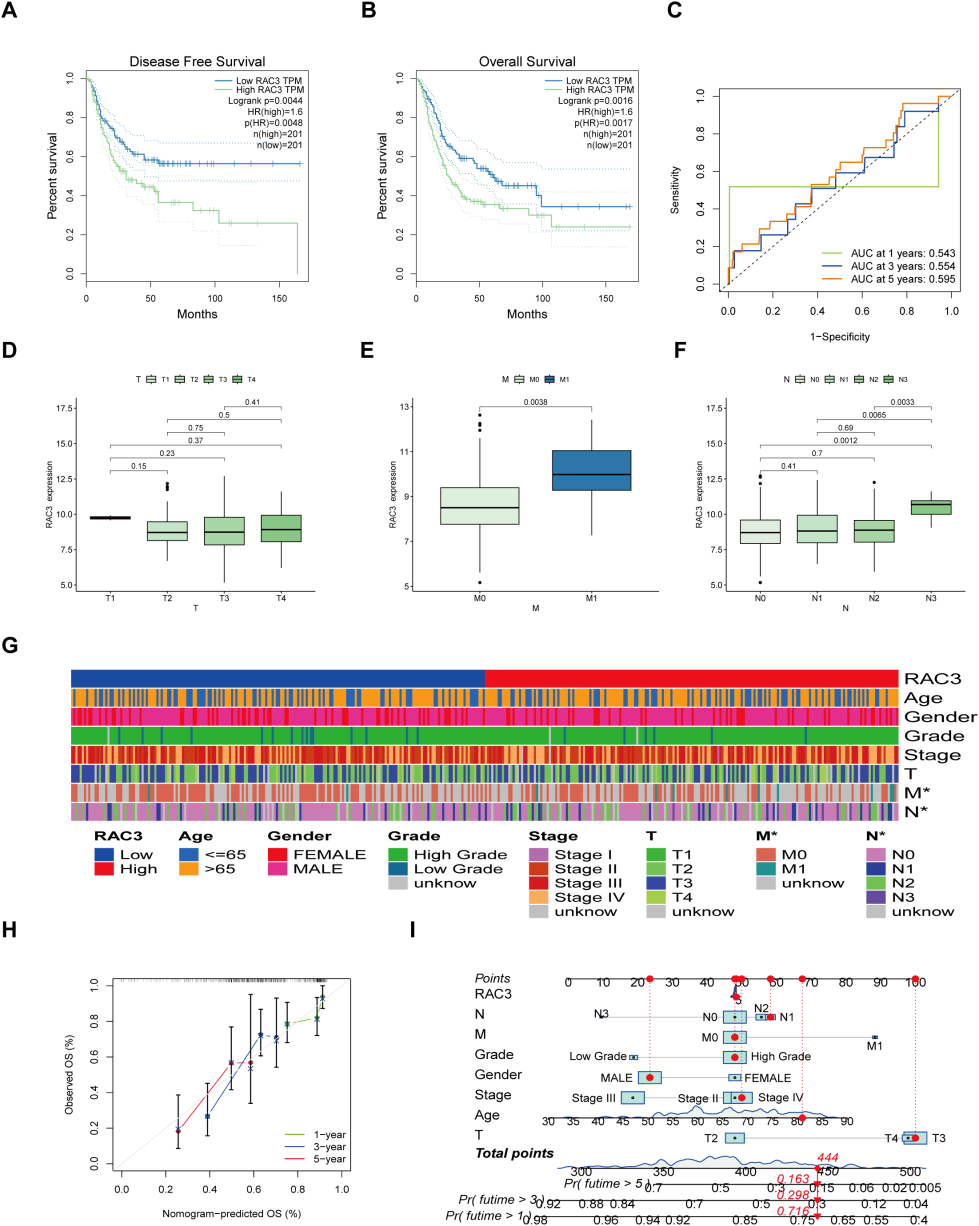
Relationships between RAC3 expression levels and patient outcomes. **(A–C)** The effects of RAC3 on disease-free survival **(A)**, overall survival **(B)** and the ROC curves are depicted **(C)**. **(D–G)** The correlation of RAC3 expression with various clinical and pathological characteristics was examined. **(H, I)** By summing the points allocated to different prognostic indicators in the nomogram scoring system, the 1-, 3- and 5-year survival probabilities were predicted.

Data from 432 bladder cancer patients within TCGA database, which included 413 tumor tissues and 19 normal tissue samples, were divided into two groups based on the median expression of the RAC3 mRNA. Kaplan–Meier survival curves confirmed that higher RAC3 expression was associated with poorer patient outcomes, specifically shorter times to disease progression and death (**Figures 2A–C**). Expanding upon these initial observations, our subsequent analysis aimed to explore the relationship between RAC3 expression and the clinical outcomes of BLCA patients. Notably, increased levels of RAC3 were correlated with several negative prognostic indicators, such as the M stage (M1 vs. M0) and the final N stage (N3 vs. N0/1/2) (**Figures 2D–G**). Furthermore, predictive models, in the form of nomograms, were developed to estimate the 1-, 3- and 5-year overall survival rates for bladder cancer patients (**Figures 2H, I**). The survival estimate was derived from the sum of the points allocated to each prognostic factor according to the nomogram’s scoring algorithm. The findings underscore the strong correlation between RAC3 expression and the prognosis, including survival rates at the specified time points, for patients with bladder cancer.

### The expression level of RAC3 is connected with immune cell infiltration and immune checkpoint mechanisms

RAC3 expression within bladder cancer tissues has been found to be linked to both the density of immune cells within the tumor microenvironment and the expression patterns of genes that are pivotal in the modulation of immune checkpoints. This association suggests that RAC3 may play a role in the interaction of tumors with the immune system, potentially influencing the effectiveness of immune checkpoint therapies. In our quest to understand how RAC3 gene expression correlates with the infiltration of immune cells in BLCA, we categorized the patient data from TCGA database into two groups using the median RAC3 mRNA expression as the dividing criterion. Postsegmentation, we employed CIBERSORT and ESTIMATE computational tools to meticulously assess the degree of immune cell infiltration within the tumor samples of the categorized patient groups. The ESTIMATE algorithm was then applied to determine the relationship between the expression levels of RAC3 and the scores indicative of immune cell infiltration. Furthermore, the MSI, TNB and TIDE algorithms were leveraged to anticipate the response to immunotherapy, particularly the impact of immune checkpoint blocking agents.

#### Analysis of RAC3 expression, immune cell infiltration and immune pathways

**Figure 3:**
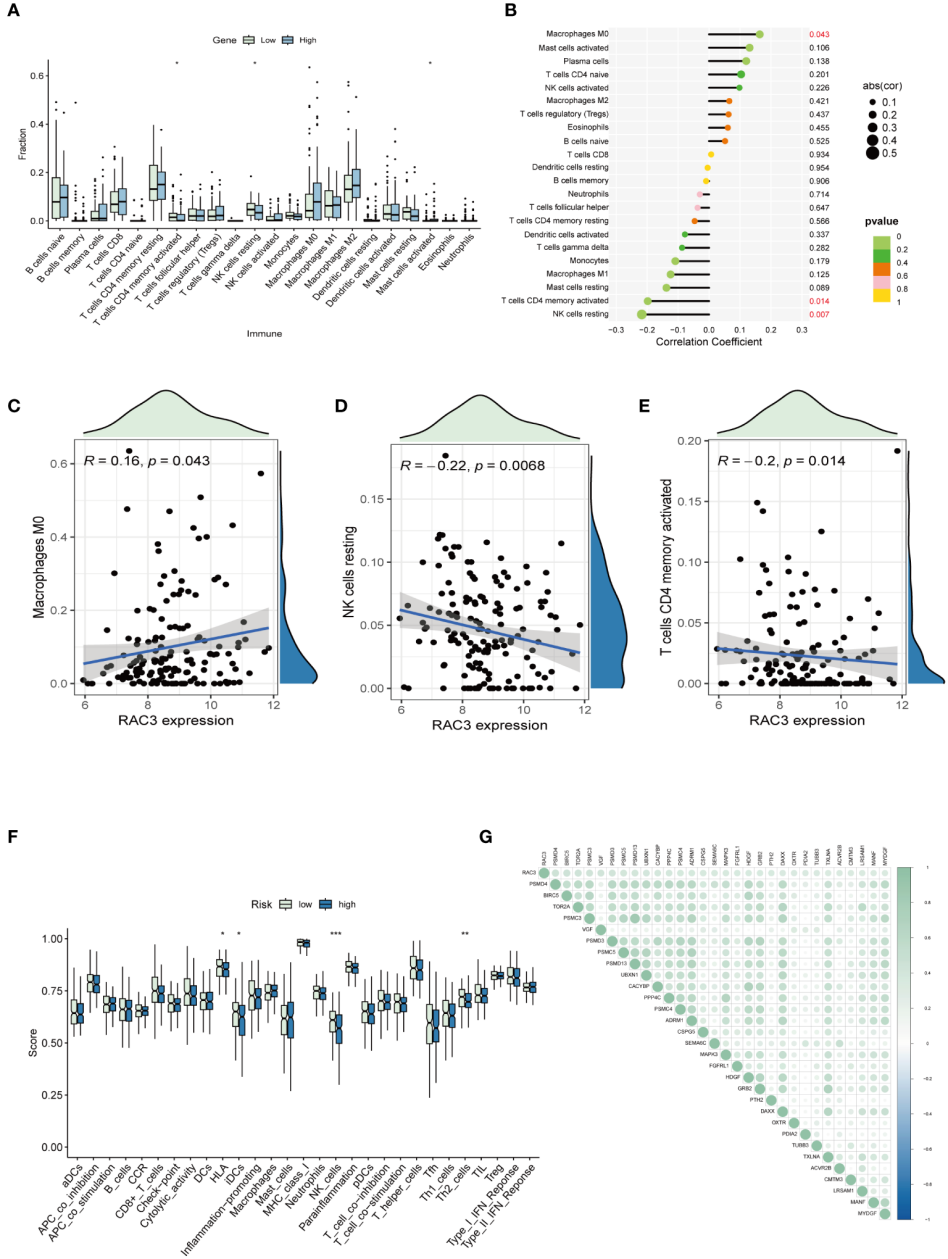
The associations of RAC3 with the presence of immune cells and immune checkpoint molecules were explored. **(A–E)** A comparative analysis was conducted to assess the variations in the infiltration levels of 22 distinct immune cell types between groups characterized by high and low levels of RAC3 expression. **(F)** A comparative analysis was conducted to assess the variation in immune pathways between groups characterized by high and low levels of RAC3 expression. **(G)** This study investigated how RAC3 expression correlated with the expression of immune checkpoint inhibitory molecules. Statistical significance is indicated by asterisks: * for p<0.05, ** for p<0.01, and *** for p<0.001.

We utilized CIBERSORT to assess the presence of 22 distinct immune cell types, including various T cells, B cells, NK cells, and myeloid cells. Only findings with a significant p value (p < 0.05) were selected for the detailed analysis. The results indicated that within the group with elevated RAC3 expression, notable increases in the numbers of M0 macrophages and activated mast cells were observed. Conversely, the group with reduced RAC3 expression presented a greater prevalence of activated memory CD4^+^ T cells and resting NK cells (**Figures 3A–E**). Furthermore, patients were stratified into high-risk and low-risk groups based on the expression levels of RAC3. An analysis was conducted to explore the correlation between RAC3 expression and various immune pathways. The findings revealed that variations in RAC3 expression were significantly associated with the presence of HLA molecules, immature dendritic cells (iDCs), NK cells, and regulatory T cells (Tregs), which are often referred to as Th2 cells in some contexts (**Figure 3F**).

#### Analysis of immune-related genes

Expanding on our prior explorations, we embarked on a correlation study examining the relationship between RAC3 and a subset of inhibitory checkpoint molecules, all of which are immune-related genes extracted from the IRG database. Spearman correlation analysis was applied to evaluate the correlation, from which we selected the initial 30 genes with the lowest P-values for detailed review. The outcome of this study highlighted a positive correlation between RAC3 and the majority of the immune checkpoints in BLCA, with PSMD4, BIRC5, and TOR2AR standing out as having the most pronounced correlation values (**Figure 3G**).

#### Analysis of RAC3 expression and TME scores

**Figure 4:**
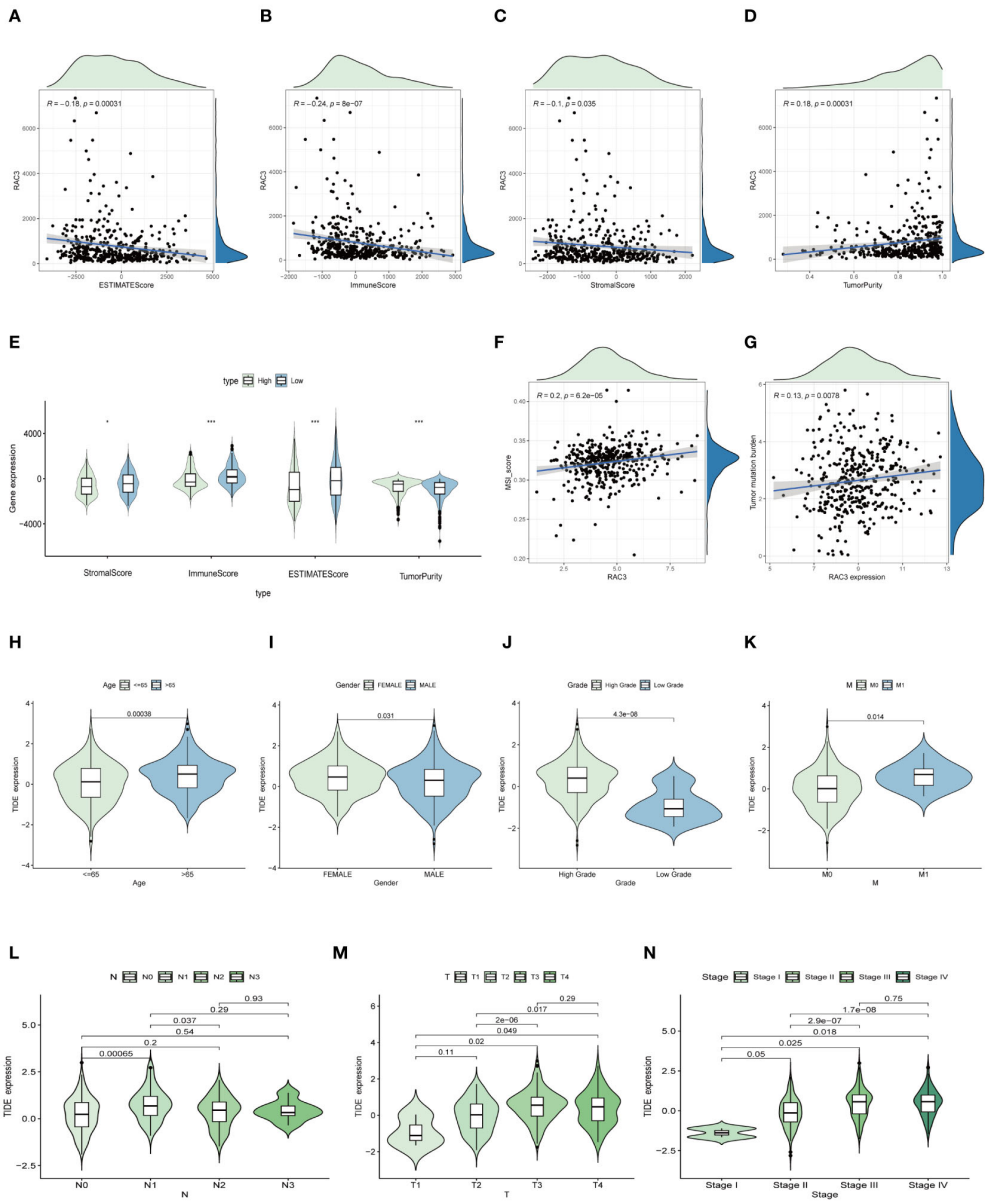
The relationship between RAC3 expression and the immune context within tumors. **(A–E)** The study analyzed how RAC3 expression correlated with scores reflecting the characteristics of the tumor microenvironment, such as the immune score and the ESTIMATE score. **(F–N)** The MSI score **(F)**, tumor mutation burden (TNB) score **(G)**, and tumor immunity estimation and direction (TIDE) model were applied to predict the potential efficacy of immunotherapy **(H–N)**. The significance of the findings is denoted by asterisks: * for p<0.05, and *** for p<0.001.

Understanding the tumor microenvironment (TME) is essential for advancing cancer research, as it dictates the trajectory of the disease and the effectiveness of treatments (29). Our study aimed to clarify the interaction between the RAC3 protein and the TME in bladder cancer (BLCA) by quantifying immune cell infiltration with the ESTIMATE algorithm (30). We calculated the stromal scores and immune scores, as well as the overall ESTIMATE score and tumor purity. Notably, the group with increased RAC3 expression had significantly lower scores in all measured categories (p=0.035, p<0.0001, and p=0.00031), and a clear positive correlation between RAC3 levels and tumor purity was observed, indicating a potential role of RAC3 in modulating the TME (**Figures 4A–E**).

#### Analysis of RAC3 expression and the response to immunotherapy

The TIDE algorithm serves as a prognostic instrument designed to assess the probable effectiveness of tumor immunotherapy, factoring in the dynamics of dysfunction and exclusion within the tumor microenvironment (TME) (31). In our investigation, the TIDE algorithm was used to predict the impact of immunotherapy mediated by immune checkpoint inhibitors. The findings indicated that female patients under the age of 65 years, with a particular emphasis on those afflicted with low-grade, early-stage cancer, presented a diminished TIDE score (**Figures 4H–N**). These findings suggest that these patients may have a greater propensity for positive responses to immunotherapy interventions. Accumulated evidence from prior research underscores the importance of the tumor mutation burden (TMB) and microsatellite instability (MSI) as key predictive indicators of a tumor’s immunological responsiveness (32). Patients characterized by a high mutational load due to the TMB and those with a high degree of MSI (MSI-H) are more likely to mount a robust immune response. Consequently, the FDA has sanctioned the use of diverse anti-PD-1 and anti-PD-L1 immune checkpoint inhibitors for therapeutic intervention in such patients (33, 34). Our study revealed a significant positive correlation between RAC3 expression and the MSI (**Figure 4F**) and TMB (**Figure 4G**) scores. This association suggests that individuals with higher RAC3 expression levels could experience greater benefits from immunotherapy. Additionally, our results introduce RAC3 as a potential novel biomarker for the prediction of the immunotherapy response.

### Potential biological functions and pathways of RAC3 in BLCA

#### Results of the GO enrichment analysis

**Figure 5:**
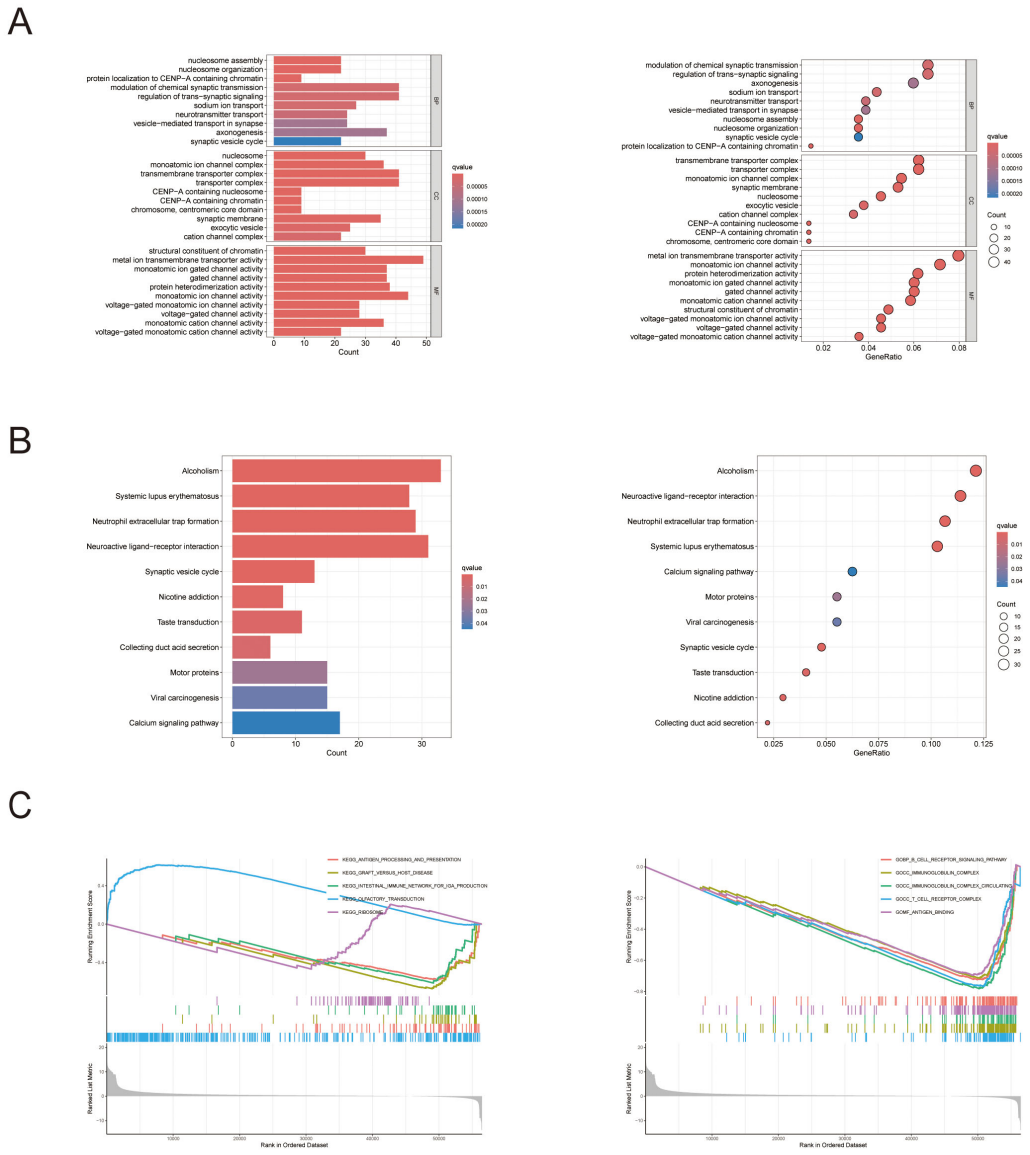
The biological functions and pathways associated with RAC3 in bladder cancer (BLCA). **(A)** The figure displays the outcomes of the Gene Ontology (GO) enrichment analysis. **(B)** The results of gene set enrichment analysis (GSEA) are presented, with a focus on the enrichment of KEGG functional pathways and **(C)** GO functional pathways.

We conducted a functional enrichment analysis to distinguish the genes that were differentially expressed across the high- and low-RAC3 expression groups, with significance defined as a log2-fold change (FC) ≥ 1 and p < 0.05. Gene Ontology (GO) analysis revealed several biological processes pivotal to cell signaling, notably those involving immune functions. The results revealed that the expression of these genes was related to the modulation of chemical synaptic transmission, the regulation of transsynaptic signaling, and nucleosome assembly and organization. Furthermore, the cellular component (CC) analysis provided an in-depth depiction of the subcellular elements implicated in gene expression, offering insights into the underlying cellular machinery associated with the observed expression patterns. We found that the expression of RAC3 was related to the transmembrane transporter complex, transporter complex and monoatomic ion channel complex. Finally, the molecular function (MF) analysis describes the biochemical activities of genes, such as metal ion transmembrane transporter activity, monoatomic ion channel activity and protein heterodimerization activity. This examination delved into the intrinsic functions of gene products at the molecular level, providing essential insights into the core biological mechanisms at play within cellular contexts (**Figure 5A**). Paralleling our approach, a gene set enrichment analysis (GSEA)-based exploration of KEGG (**Figure 5B**) and GO (**Figure 5C**) pathways revealed the enrichment of the immune network for IgA production, the B- and T-cell receptor signaling pathways and the immunoglobulin complex. After synthesizing our observations, the data collectively suggest that increased RAC3 expression in BLCA is correlated with intensified immune responses. This correlation may indicate that RAC3 is a promising adjunctive therapeutic target for patients who have demonstrated resistance to conventional anti-PD-1/PD-L1 treatment modalities.

#### RAC3 promotes BLCA cell proliferation, migration and invasion

**Figure 6:**
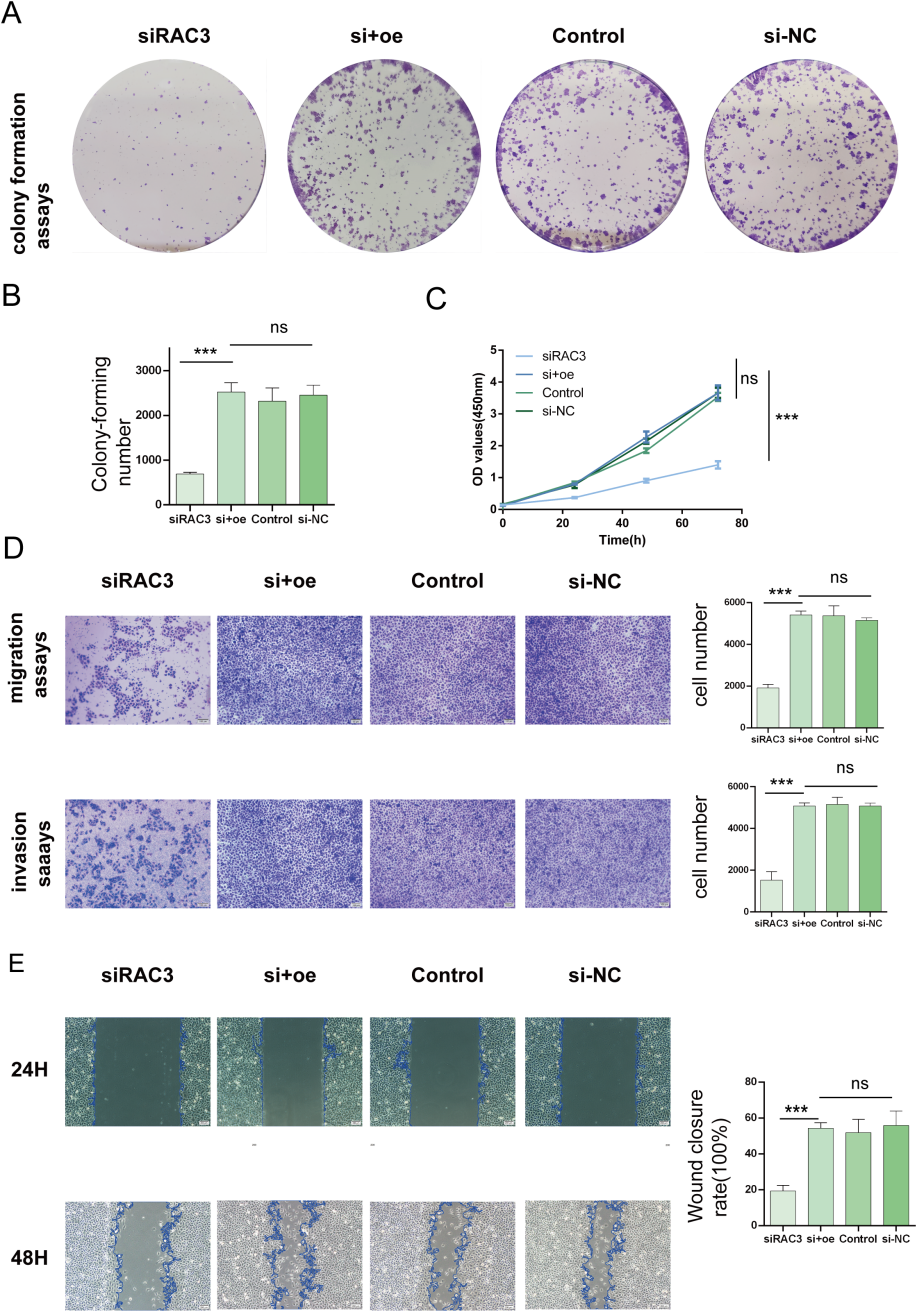
Impacts of RAC3 on the proliferation, migration, and invasion of bladder cancer-derived 5637 cells **(A, B)** A colony formation assay was conducted to assess the effect of RAC3 on cell proliferation. **(C)** The CCK-8 assay was also utilized to further analyse the proliferation capabilities of cells with different RAC3 expression levels. **(D)** Transwell assays were used to evaluate the invasive and migratory abilities of the cells under the influence of RAC3. **(E)** A wound healing assay was conducted to measure the migration behavior of the cells, providing additional insights into their motility. The experiment was repeated at least 3 times. The significance of the differences observed is indicated by asterisks: *** for p<0.001. ns for P>0.05.

**Figure 7:**
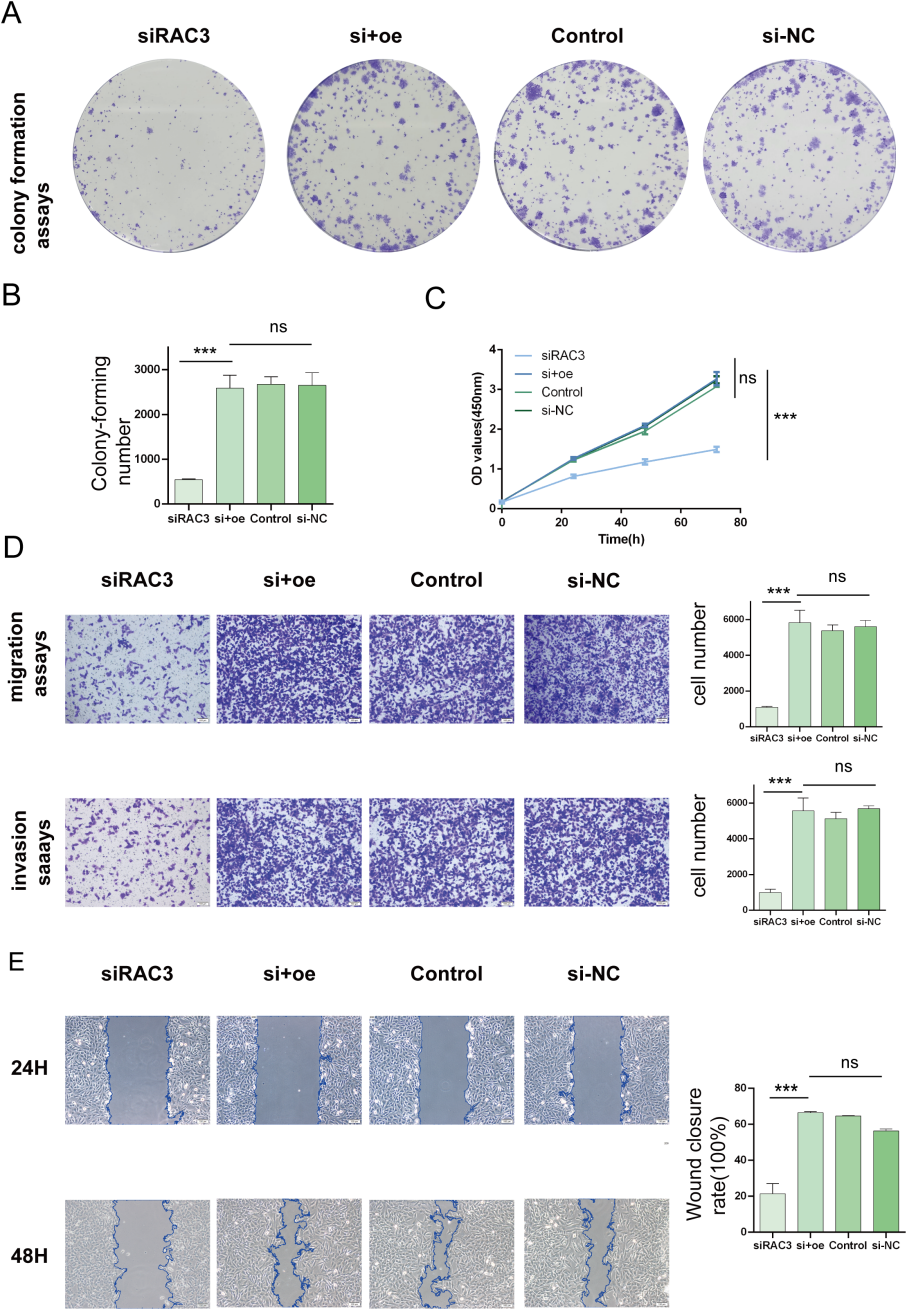
Impacts of RAC3 on the proliferation, migration, and invasion of bladder cancer-derived T24 cells. **(A, B)** A colony formation assay was conducted to assess the effect of RAC3 on cell proliferation. **(C)** The CCK-8 assay was also utilized to further analyse the proliferation capabilities of cells with different RAC3 expression levels. **(D)** Transwell assays were used to evaluate the invasive and migratory abilities of the cells under the influence of RAC3. **(E)** A wound healing assay was conducted to measure the migration behavior of the cells, providing additional insights into their motility. The experiment was repeated at least 3 times. The significance of the differences observed is indicated by asterisks: *** for p<0.001. ns for P>0.05.

In an effort to decipher the potential interplay between RAC3 expression levels and the aggressive behavior of bladder cancer cells, we strategically utilized siRNAs to silence RAC3 in BLCA 5637 cells and T24 cells. The experimental results revealed the substantial suppression of cancer cell characteristics such as cell proliferation, migratory patterns, invasiveness, and the ability to form colonies upon RAC3 knockdown. This inhibition is theorized to be associated with the reduced capacity of the cells to withstand aggressiveness. We further quantified the clonal potential of the cells using a colony formation assay (**Figures 6A, B**, **7A, B**). Additionally, the CCK-8 assay was utilized to evaluate the influence of RAC3 on cell proliferation (**Figures 6C**, **7C**). The results indicated that the introduction of si-RAC3 markedly decreased the cellular proliferation rate compared with that of both the si-NC group and the untreated controls. Moreover, we assessed the impact of RAC3 on cell invasiveness and motility using a Transwell assay (**Figures 6D**, **7D**) and a wound healing assay to measure migration capacity (**Figures 6E**, **7E**). Knocking down RAC-3 while simultaneously overexpressing the gene can reversed the reductions in cell proliferation, migration, and invasion capabilities caused by the decreased expression of RAC-3. By restoring RAC-3 expression in cells wherein which it has been knocked down, researchers can determine whether the observed effects on cell behavior are indeed attributable to the manipulation of RAC-3 levels. If the phenotype is reversed upon overexpression, this finding strengthens the evidence that RAC-3 plays a critical role in these cellular processes. Both assays consistently indicated that the attenuation of RAC3 expression substantially compromised the invasive and migratory capabilities of bladder cancer cells. Taken together, these observations highlight the putative role of RAC3 as a central modulator of the proliferative, invasive, and metastatic activities of bladder cancer, suggesting its potential as a novel therapeutic target for BLCA.

### Exploring the link between RAC3 and drug resistance in bladder cancer

**Figure 8:**
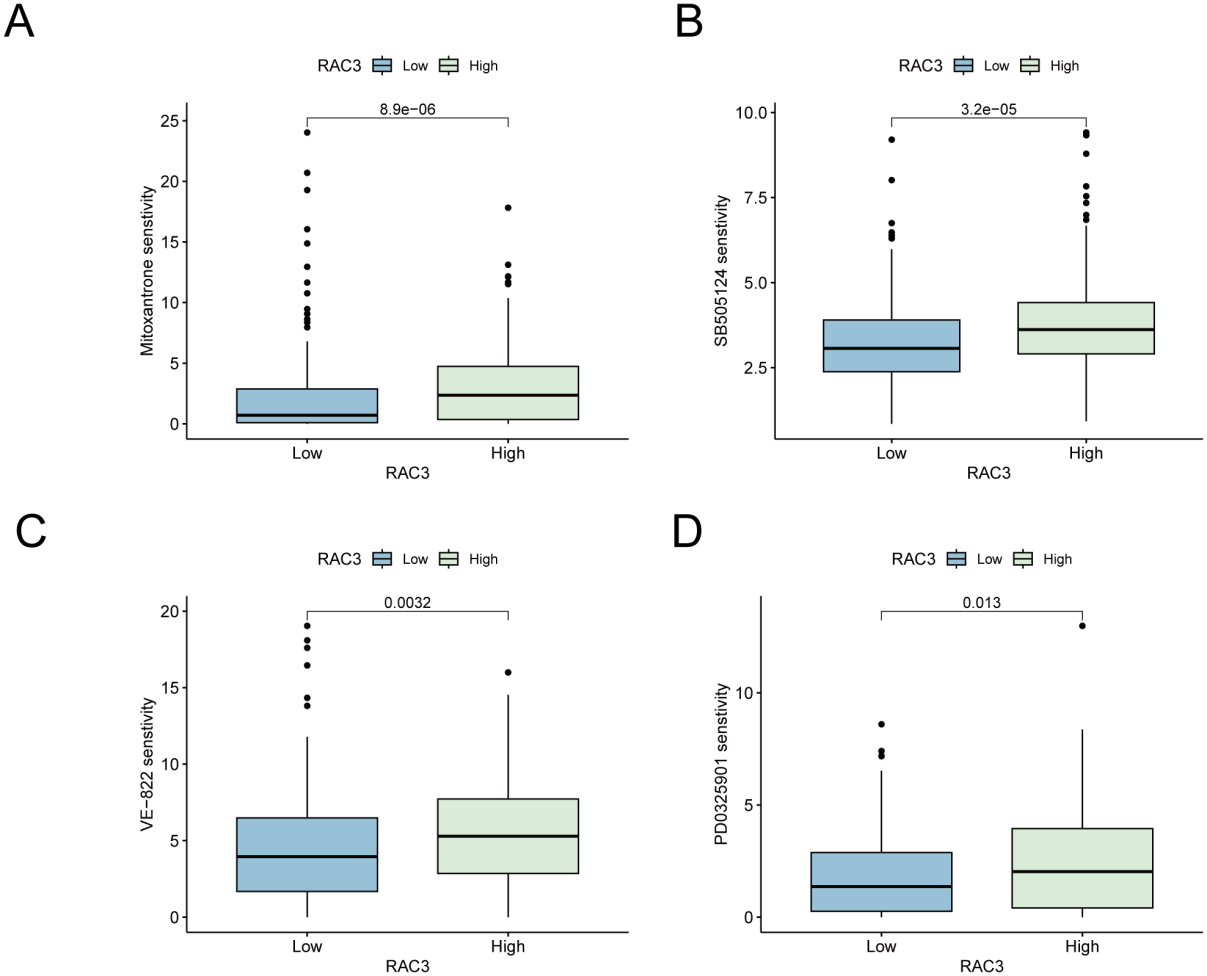
The expression of RAC3 is implicated in the development of resistance to chemotherapy and immunotherapy drugs. **(A)** senstivity for drug mitoxantrone. **(B)** senstivity for drug SB505124. **(C)** senstivity for drug VE-822. **(D)** senstivity for drug PD0325901.

We stratified patients into high- and low-expression cohorts using the median RAC3 expression level as the cut-off to elucidate the correlation between RAC3 expression and chemoresistance in bladder cancer. The IC50 value, an established metric of drug sensitivity with an inverse relationship to sensitivity, was utilized to assess the drug response. We harnessed the OncoPredict analytical package to evaluate the drug resistance profiles of the patient groups (35). the oncoPredict package, which we utilized for assessing patient sensitivity to 196 chemotherapeutic drugs based on IC50 values, operates on the principle that a higher IC50 value corresponds to a higher sensitivity score. This means that a higher score indicates lower sensitivity, or increased drug resistance, to the respective compound. Our analysis indicated that patients exhibiting elevated RAC3 expression displayed reduced sensitivity to a subset of therapeutic agents, notably mitoxantrone (**Figure 8A**), SB505124 (**Figure 8B**), VE-822 (**Figure 8C**) and PD0325901 (**Figure 8D**). These findings suggest that increased RAC3 levels may contribute to the acquisition of resistance to a spectrum of treatments, including chemotherapy, targeted therapies, and immunotherapies, within bladder cancer. This refined investigation provides reference value for further exploration into the role of RAC3 as a potential biomarker for chemoresistance and its implications for personalized therapeutic strategies in bladder cancer management.

## Discussion

According to the International Agency for Research on Cancer (IARC) report of the World Health Organization in 2020 (36), 570,000 new cases of bladder cancer occurred globally, accounting for 3% of the total incidence of cancer, with 210,000 deaths. In men in particular, it is the sixth most common malignant tumor. Bladder cancer treatments include surgical treatment, chemotherapy, radiotherapy, and targeted therapy. However, due to the lack of early diagnostic methods, patients often present at an advanced stage when they seek medical attention due to symptoms. Even after standardized surgical treatment, recurrence and metastasis are still prone to occur. For these patients, platinum-based doublet therapy is the first-line treatment, but drug resistance is common. Patients are insensitive to second-line treatments, and the treatment efficacy is poor. With the advent of the immunotherapy era represented by PD-1/PD-L1 inhibitors, research has shown that immunotherapy can benefit some bladder cancer patients, but it is only effective for a small number of patients, especially those with high expression of PD-L1. Therefore, finding more effective immunotherapy targets and therapeutic approaches or reducing the occurrence of resistance to first-line treatments is highly important for improving patients’ clinical outcomes. Therefore, our team has identified a novel immunotherapeutic target with potential value, with the aim of achieving this objective.

RAC3, a member of the Ras protein family, significantly influences a range of critical biological processes, such as adhesion, morphology, migration, and the cell cycle, underscoring its importance in a diverse array of cell types. Extensive research has implicated RAC-3 in the genesis of multiple cancers, confirming its classification as a broad-spectrum oncogene (37, 38). However, the precise mechanisms through which it modulates tumor progression, particularly in bladder cancer, are not fully understood and are understudied. Our research revealed that RAC3 is markedly overexpressed in bladder cancer tissues and cells compared with their normal counterparts and that this overexpression is linked to the severity of malignant characteristics and an unfavorable prognosis for patients. By employing RNA interference to knock down RAC3 expression, we observed notable suppression of the proliferative, migratory, and invasive capabilities of bladder cancer cells. The effect of siRNAs on RAC-3 expression and cell malignant progression can be reversed by transfecting an overexpression plasmid. These findings underscore the pivotal role of RAC3 in the malignant progression of bladder cancer. This discovery highlights RAC3 as a promising candidate for targeted therapies, warranting further exploration as a potential therapeutic strategy. The development of drugs aimed at modulating RAC3 activity could offer a novel approach to combat bladder cancer, particularly for patients facing resistance to existing treatments. This avenue of research holds great potential for improving clinical outcomes and providing new hope for individuals battling this aggressive disease. However, the precise mechanisms by which RAC3 contributes to the malignancy of tumor cells remain unclear. Many immune checkpoints regulate the immune microenvironment, thereby controlling the immune evasion of tumor cells and leading to disease progression. Therefore, we will explore whether RAC3 can modulate the immune microenvironment in our next phase of research.

The immune microenvironment is a pivotal determinant of cancer patient outcomes, significantly influencing responses to immunotherapy and potentially contributing to the development of resistance to both chemotherapy and immunotherapy (39, 40). A nuanced understanding of the tumor immune microenvironment, with a particular focus on the patterns of immune cell infiltration, is essential for elucidating the aetiology and progression of bladder cancer. Prior studies have demonstrated that elevated RAC3 levels in epithelial cancer tissues are inversely linked to the infiltration of CD8+T cells, thereby orchestrating a microenvironment that suppresses the immune response and targeting RAC3 offers a strategic approach to reconfigure the immune microenvironment, thereby amplifying the potency of immune cells in combating tumor growth (41). Our research revealed that RAC3 expression modulates the signaling pathways of B- and T-cell receptors, which are integral to the immune response, in bladder cancer. The presence of immune cells within the tumor microenvironment is a critical determinant of the effectiveness of the immune response. Furthermore, we observed a correlation between RAC3 expression and the IgA-producing immune network, as well as the accumulation of immunoglobulin complexes. Accumulating evidence suggests that IgA and other immunoglobulins play a significant role in the interplay between cancer development and the immune response, potentially influencing the efficacy of immunotherapies and the body’s ability to combat tumor growth (42, 43). This insight into the multifaceted role of RAC3 in the immune landscape of bladder cancer may provide novel avenues for therapeutic intervention and a better understanding of the complex dynamics at play in cancer immunology.

We identified an increased immune score in patients with elevated RAC3 expression, which was correlated with the infiltration of diverse immune cells and immune pathways. Intriguingly, our findings suggest that female patients under 65 years of age with low-grade, early-stage cancer exhibit reduced TIDE scores, indicating potential responsiveness to immune checkpoint inhibitors. This insight provides a rationale for the potential therapeutic utility of RAC3 targeting in patients who are refractory to conventional anti-PD-1/PD-L1 therapies. In conclusion, the collective observations from our study underscore the potential of RAC3 as a valuable therapeutic target in bladder cancer, particularly for those who may not benefit from existing immunotherapies. The role of RAC3 in the immune microenvironment represents a promising avenue for the development of novel treatment strategies that could enhance the efficacy of immunotherapy and improve patient outcomes. While immunotherapy alone is effective for only a subset of patients, the exploration of combination therapies remains a mainstream direction in cancer treatment. The conclusion that immunotherapy combined with chemotherapy can enhance therapeutic efficacy has been validated in numerous clinical studies. With this result in mind, we are further investigating whether RAC3 regulates the development of chemoresistance in the hopes of providing a theoretical foundation for combination therapies. Our aim is to elucidate the role of RAC3 in chemotherapy resistance, which could inform more effective treatment strategies that combine the strengths of immunotherapy with the targeted action of chemotherapeutic agents.

Platinum-based chemotherapy regimens are a mainstay in the treatment of bladder cancer; however, the propensity for these tumors to develop resistance presents a formidable clinical obstacle. The mechanisms behind this resistance remain largely elusive. Notably, heightened expression of RAC3 has been identified in BLCA tissues that exhibit drug resistance, where it is implicated in augmenting chemoresistance through the PAK1-ERK1/2 signaling pathway (44). Our research revealed a correlation between elevated RAC3 levels and diminished sensitivity to specific therapeutic agents, such as mitoxantrone, a chemotherapeutic agent for bladder cancer, as well as SB505124 and VE-822. SB505124, when coadministered with cisplatin, can enhance the tumor response to cisplatin, potentially reducing the need for prolonged cisplatin therapy (45). VE-822, an ATM/ATR kinase inhibitor, has been shown to inhibit cell proliferation, colony formation, and migration by suppressing p-ATR expression, thereby disrupting DNA repair mechanisms and potentiating the effects of cisplatin on a nude mouse xenograft model (46). Additionally, PD0325901, an ERK inhibitor, has been shown to augment the efficacy of PD-1 inhibitors (47). These findings suggest that the overexpression of RAC3 is associated with an increased risk of resistance to cisplatin and immune checkpoint inhibitors, potentially accounting for the poor prognosis of patients with high RAC3 expression. Consequently, immunotherapy strategies that target RAC3 could represent a synergistic therapeutic approach when combined with cisplatin chemotherapy, potentially enhancing treatment efficacy. Moreover, for patients who are resistant to cisplatin and PD-1/PD-L1 inhibitors, RAC3-targeted therapies may be promising for reversing drug resistance and improving survival outcomes.

## Conclusion

I In this comprehensive study, we explored the intricate role of Ras-related C3 (RAC3), a pivotal member of the Rho subfamily of Ras proteins, in the modulation of bladder cancer (BLCA) progression. Our research highlights the significant association of RAC3 with key biological processes, including the promotion of cell proliferation, migration, invasion, and somatic growth, as well as its influence on the tumor microenvironment (TME) and immune response. Our findings reveal that increased RAC3 expression is linked to an aggressive phenotype of BLCA, with implications for chemoresistance and immune evasion. The molecular mechanisms implicated in these effects suggest that RAC3 modulates the immune landscape within the TME, potentially through its interaction with nuclear receptors and transcription factors, thereby influencing the expression of chemokines and immune cell infiltration patterns. In conclusion, our research identifies RAC3 as a multifaceted regulator in BLCA, with potential as a novel target for immunotherapeutic interventions. The elucidation of the role of RAC3 in the immune microenvironment and chemoresistance mechanisms opens avenues for the development of personalized medicine approaches, which may ultimately enhance the efficacy of therapeutic strategies and improve clinical outcomes for patients with high-risk BLCA. Further in-depth investigations are warranted to dissect the complex interplay of RAC3 in tumor biology and to harness its full potential in bladder cancer management.

## Data Availability

The original contributions presented in the study are included in the article/**Supplementary Material**. Further inquiries can be directed to the corresponding author/s.
