# Supplementary material for: Revealing the mechanisms of RAC3 in tumor aggressiveness, the immunotherapy response, and drug resistance in bladder cancer
Source: Front Oncol. 2024 Sep 16;14:1466319. doi: 10.3389/fonc.2024.1466319 (PMC11441374; doi:10.3389/fonc.2024.1466319)
Supplement: Supplementary file 1 [file Table1.docx]

| RAC3-si-3 | F: 5’- CAACUACUCUGCCAACGUGAUTT-3’  R: 5’- AUCACGUUGGCAGAGUAGUUGTT-3’ |
| --- | --- |
| RAC3-si-2 | F: 5’-CUUCGAGAAUGUUCGUGCCAATT-3’  R: 5’-UUGGCACGAACAUUCUCGAAGTT-3’ |
| RAC3-si-1 | F: 5’-CCGGGAGAUUGGCUCUGUGAATT-3’  R: 5’-UUCACAGAGCCAAUCUCCCGGTT-3’ |
| RAC3 | F: 5’-GCCTCCGACGTCTGCATAGAAC-3’  R: 5’-GCGAAGAACTCGACGGCAGCAT-3’ |
| GAPDH | F: 5’-ACCACAGTCCATGCCATCAC-3’  R: 5’-TCCACCACCCTGTTGCTGTA-3’ |

S1: The primer sequences
