# Supplementary figures and images for: Revealing the mechanisms of RAC3 in tumor aggressiveness, the immunotherapy response, and drug resistance in bladder cancer
Source: Front Oncol. 2024 Sep 16;14:1466319. doi: 10.3389/fonc.2024.1466319 (PMC11441374; doi:10.3389/fonc.2024.1466319)

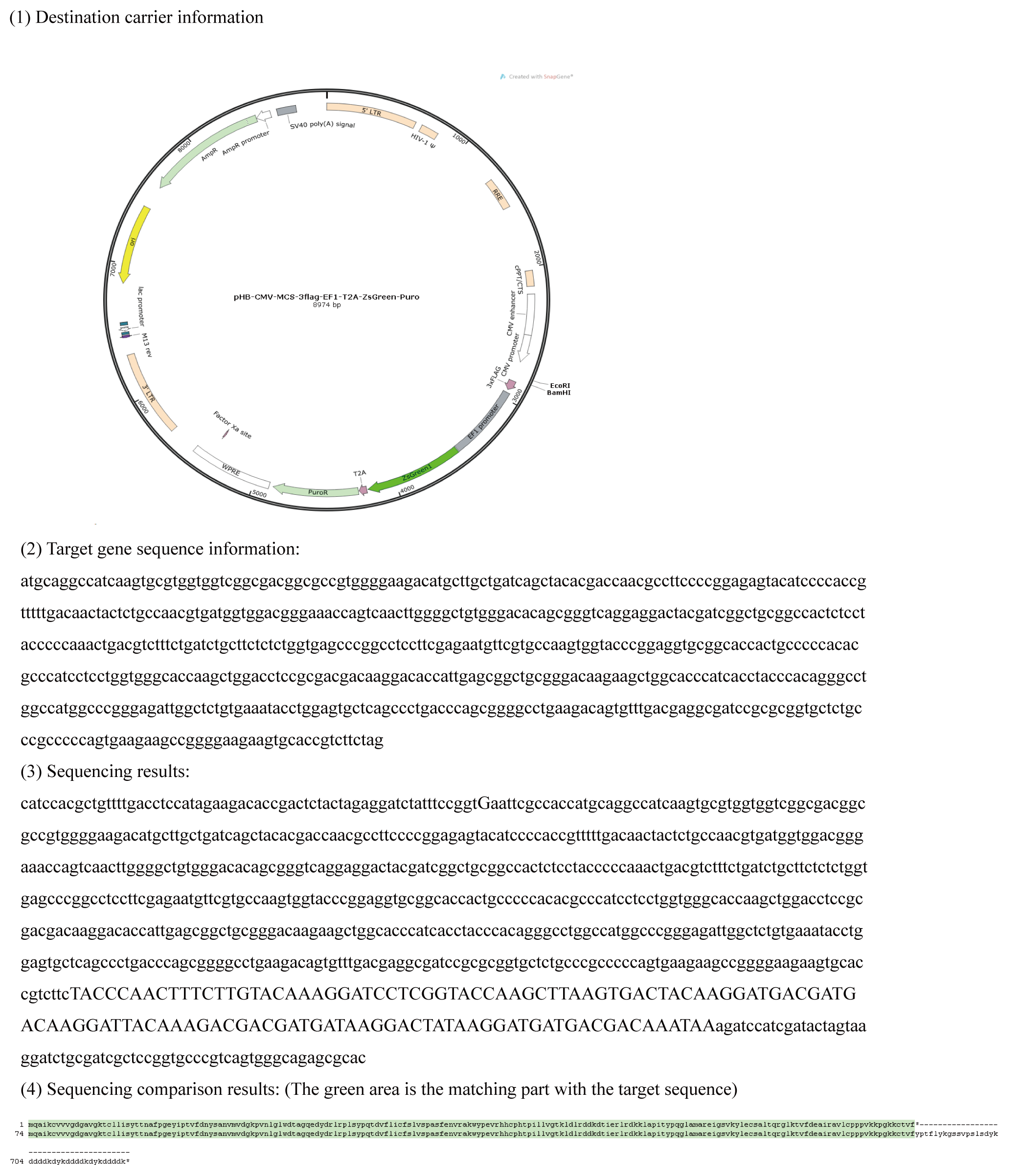

Supplement: Supplementary file 2 [file Image1.tif]
